# The TBXT Gene and Brachyury Protein Are Differentially Expressed During the Early Embryonic Development of Hu and Hulunbuir Sheep

**DOI:** 10.3390/life15101560

**Published:** 2025-10-05

**Authors:** Daqing Wang, Yifan Zhao, Guifang Cao, Jiajia Zhang, Caiyun Wang

**Affiliations:** 1 College of Veterinary Medicine, Inner Mongolia Agricultural University, Hohhot 010011, China; wangdaqing050789@126.com (D.W.); yifanz311@163.com (Y.Z.);; 2 Animal Embryo and Developmental Engineering Key Laboratory of Higher Education, Institutions of Inner Mongolia Autonomous Region, Hohhot 010011, China; 3 Inner Mongolia Autonomous Region Key Laboratory of Basic Veterinary Medicine, Hohhot 010011, China; 4 College of Life Sciences, Inner Mongolia University, Hohhot 010021, China

**Keywords:** TBXT gene, Hulunbuir short-tailed sheep, lake sheep, in situ hybridization, immunofluorescence

## Abstract

In this study, SweAMI FISH fluorescence in situ hybridization and immunofluorescence were used to analyze the spatiotemporal expression characteristics of the TBXT gene and Brachyury protein in 16-day-old Hulunbuir sheep embryos and 19-day-old Hu sheep embryos and to explore their regulatory effects on the development of sheep tails and related organs. The study showed the following: At 16 days of embryonic age, the TBXT gene was concentratedly expressed in the heart, somites, neural tube, and mesonephros of both sheep breeds; at 19 days of embryonic age, it was concentratedly expressed in the limb ectoderm and tail bud of Hulunbuir sheep, and in the midgut and tail bud of Hu sheep. At 16 days of embryonic age, the BRACHYURY protein was concentratedly expressed in the neural tube, somites, brain vesicles, and mesonephros of both sheep breeds; at 19 days of embryonic age, it was concentratedly expressed in the heart and hindgut of Hulunbuir sheep, and in the tail bud and hindgut of Hu sheep. In summary, this shows that there are differences in the temporal and spatial expressions of the TBXT gene and BRACHYURY protein between the two sheep breeds. There are also site-specific and time-specific differences in the regulation of the above genes and proteins during tail and related organ development between the two breeds, which confirms that the molecular regulation pathways of tail and related organ development are different between the two breeds. This study provides an experimental basis for screening molecular markers related to goat tail development and breed improvement.

## 1. Introduction

Hulunbuir sheep is a meat-type sheep breed distributed in the Hulunbuir region of Inner Mongolia Autonomous Region, China. It has been developed through long-term artificial selection and natural selection and belongs to the Mongolian sheep lineage [1]. Among them, the Hulunbuir short-tailed sheep, also known as Xinihe sheep due to its primary habitat in the Xinihe River basin, is also nicknamed Buryat sheep as it is raised by the local Buryat Mongolian tribe [2]. This breed is characterized by a strong and well-proportioned physique, with a moderately sized head, large drooping ears, a slightly protruding bridge of the nose, a short and thick neck, and sturdy limbs with plump thigh muscles. It has a rounded rib cage, a straight back and loin, well-developed hindquarters, a broad and deep body, and a flat, wide rump that is slightly rectangular in shape. The coat consists of white heterogeneous wool, with colored wool on the head and parts below the carpal and hock joints. Some rams have horns, while ewes are polled. Its most distinctive feature is the tail: the withers are slightly lower than the height of the sacrum and slope forward, and the tail is short and broad. This short-tailed phenotype reduces entanglement obstacles when moving in grassland shrubs and minimizes the risk of tail frostbite in severe winter, making it highly adaptable to the complex environment of the northern grasslands [2].

Hu sheep belong to the genus Ovis of the family Bovidae, order Artiodactyla, class Mammalia. Originating from the Taihu Lake basin, they are a national first-class protected local livestock breed in China [3]. This breed is less affected by climate and possesses advantages such as rapid growth and development, high-quality meat, stable production performance, and strong adaptability. It is also a world-renowned multi-fetal sheep breed [3,4]. Hu sheep have a medium-sized physique, with both rams and ewes being polled. They have a narrow and long head, a protruding bridge of the nose, most with drooping ears, a slender neck, a narrow and long body, a straight back and loin, and a slightly drooping abdomen. The coat is white, with coarse, sparse, and short wool on the abdomen, and the body is sturdy [4]. Its tail morphology is significantly different from that of Hulunbuir short-tailed sheep: it is flat and rounded with a naturally upturned tip. This tail shape retains the function of energy storage while avoiding hindrance to daily activities due to excessive tail weight, making it suitable for intensive breeding models in agricultural areas [4].

The TBXT gene is a key regulatory gene in embryonic development. It is highly expressed in the primitive streak, tail bud, and notochord of early mouse embryos, and its core function is to activate specific genes involved in mesoderm development [5]. Abnormal expression of the TBXT gene during embryonic development due to mutations or deletions may lead to incomplete notochord development, thereby causing tail malformations in animals. This suggests that the TBXT gene may be a key molecular mechanism influencing the formation of the short-tailed phenotype in animals [5]. To verify this hypothesis, In 2018, Zhi Dafu conducted whole-genome resequencing of Hulunbuir short-tailed sheep and performed sequence comparison analysis with Barga sheep, which differ only in tail morphology [1]. The results revealed that a mutation (c.G334T) in the TBXT gene, which is associated with vertebral development, may be an important factor contributing to the abnormal tail phenotype of Hulunbuir short-tailed sheep. However, this study only focused on gene sequence mutations in Hulunbuir short-tailed sheep and did not investigate the spatiotemporal expression dynamics of the TBXT gene during early embryonic development (e.g., changes in expression location and level across different embryonic ages and tissues). Additionally, no comparison was made with other breeds with significantly different tail morphologies (such as Hu sheep), making it impossible to explain why this mutation causes a short tail in Hulunbuir short-tailed sheep while Hu sheep, which lack this mutation, exhibit a flat, rounded, and upturned tail. Furthermore, Tian Zhipeng (2018) used RNA in situ hybridization probe technology to study 11.5 dpc embryos of normal mice and mice with TBXT gene mutations, confirming high expression of the TBXT gene in the embryonic head and tail bud [6,7]. However, this study used mice as the research object, and species differences (mouse vs. sheep) may lead to variations in gene function and expression patterns. Moreover, it did not involve expression differences between different sheep breeds, making it difficult to provide targeted references for studying the molecular mechanism of sheep tail development.

BRACHYURY protein is the first transcription factor identified in the T-box family. Discovered and cloned in mice by Herrmann and colleagues in 1990, the T-box family currently includes 18 members, all of which contain evolutionarily highly conserved sequences [8]. In mice, the gene encodes a DNA-binding protein consisting of 436 amino acids. The 229 amino acids near the N-terminus form a T-domain, which can bind to a 20-base palindromic target sequence (T-binding site) [9]. This protein is not involved in the formation of the anterior part of the embryo but is crucial for posterior notochord development and tail axis elongation. It is expressed in the primitive streak, tail bud, and notochord of early mouse embryos and is an essential component for mesoderm formation and tail development. It not only activates specific genes related to mesoderm development but also participates in morphogenesis, cell adhesion, polarity regulation, cell differentiation, and cell migration induction, influencing embryonic development through multiple pathways [10]. The absence of functional BRACHYURY protein leads to abnormal embryonic development in mice, with incomplete notochord differentiation, which in turn causes abnormal development of the neural tube, somites, and vertebral bodies. Heterozygous mice exhibit a short-tailed phenotype due to incomplete tail structure [10]. However, the expression differences of this protein in early embryos of sheep breeds with different tail morphologies and its regulatory mechanism associated with tail development remain unclear.

During sheep embryonic development, the formation of key structures is closely related to gene and protein expression. Studies have shown that sheep develop their first pair of somites on the 15th day after mating; at this stage, the somites appear as solid cell clusters, and myocoeles can be observed in the radial somites. From days 8 to 12, the embryo develops to the gastrulation stage, with spherical blastocysts and oblate embryonic knots [11]. On day 12, blood islands begin to form in the lateral mesoderm on the dorsal side of the primitive gut; subsequently, the mesoderm extends to the extraembryonic region, forming blood islands in the visceral mesoderm of the extraembryonic region on the surfaces of the yolk sac and allantois [11]. On day 14, the brain vesicles are long and spindle-shaped, and the embryonic disc grows rapidly. A primitive streak forms in the middle of the ectoderm, laying the foundation for the formation of the embryonic axis primordium. As the embryo develops, a notochord gradually forms, and the appearance of the neural plate creates conditions for the formation of ganglia and somites [11]. On day 16, the embryonic disc is wide at the head and tail and narrow in the center; the neural folds along the embryonic axis begin to fuse to form the neural tube, and 6 pairs of somites appear, giving the embryo a shoe-sole shape. Mesoderm emerges lateral to the somites, and the primitive knot, primitive pit, primitive streak, and amniotic knot remain. The allantoic bud, located on the posterior ventral surface of the embryonic disc, is hemispherical [12]. At this stage, the primitive gut is a long tube divided into three segments: foregut, midgut, and hindgut. The anterior part of the embryo forms primordia of structures such as the mouth, pharynx, and lung buds, while the hindgut extends toward the tail of the embryo to form primordia of the rectum and cloaca. The anterior neuropore of the neural tube remains unclosed [12]. On day 17, the anterior segment of the neural tube differentiates into forebrain vesicles, midbrain vesicles, and rhombencephalic vesicles [13]. On day 18, the forebrain vesicles further differentiate into telencephalic vesicles and diencephalic vesicles, the midbrain vesicles continue to develop, and the rhombencephalic vesicles differentiate into metencephalic vesicles and myelencephalic vesicles (a total of 5 brain vesicles). Meanwhile, the diencephalon protrudes bilaterally to form optic vesicles [13]. On day 21, the embryo assumes a C-shape; sclerotome cells migrate toward the notochord, gill slits degenerate, and a blind tube (the gallbladder) extends from the endoderm behind the stomach bud. On day 30, the stomodeum connects with the foregut, and primordia of the eyes, ears, nose, and lips gradually form [11]. Among these stages, days 16 and 19 are critical periods for the formation of key structures closely associated with tail development, such as somites, neural tubes, and brain vesicles. The expression patterns of the TBXT gene and BRACHYURY protein during these stages may play a decisive role in the subsequent differentiation of the tail and related organs, as well as the formation of breed-specific phenotypes.

Fluorescence in situ hybridization (FISH) is a non-radioactive molecular cytogenetic technique developed in the late 1980s based on radioactive in situ hybridization [14]. Currently, this technique is widely used in fields such as animal and plant genome structure research, chromosome fine structural variation analysis, cancer genetics, and genome evolution research. Among FISH-based methods, SweAMI FISH adopts the principle of nucleic acid exchange hybridization. By attaching a circular sequence to the tail of a specific probe sequence, secondary hybridization of the circular sequence generates more labels. Compared with traditional probes (which have limited labels and low sensitivity), SweAMI FISH improves sensitivity by more than 30 times, specifically enhances signals, and enables accurate localization and quantification of target gene–probe hybrids [15]. Immunofluorescence (IF) technology is an important immunochemical technique that can detect and localize various antigens in different types of tissues prepared from various cells. Compared with immunohistochemical methods using various microscopy techniques, IF has significantly higher sensitivity and signal amplification capabilities. Although fluorescent dyes can bind to antibody globulins (for detecting or localizing various antigens) or other proteins (for detecting or localizing antibodies), fluorescent antigen technology is rarely used in practical work. Therefore, it is commonly referred to as fluorescent antibody technology or immunofluorescence technology [16].

Given that existing studies have not clarified the spatiotemporal expression differences of the TBXT gene in early sheep embryos, nor have they determined the expression characteristics of BRACHYURY protein in sheep breeds with different tail morphologies, it remains impossible to explain the molecular regulatory differences in tail development between these two breeds. In this study, embryos of Hulunbuir short-tailed sheep and Hu sheep at 16 and 19 days of development (critical stages) were selected as research objects. Combining SweAMI FISH technology (for accurate localization and quantification of the TBXT gene) and immunofluorescence technology (for detecting BRACHYURY protein expression), the spatiotemporal expression characteristics of the TBXT gene and BRACHYURY protein were analyzed [17,18]. The aim was to reveal the molecular mechanism of sheep tail development and breed genetic differences, thereby laying an experimental foundation for studying the molecular mechanism of sheep tail development and screening breed-specific genetic markers.

## 2. Materials and Methods

### 2.1. Materials

Hulunbuir and Hu sheep were used in this experiment, and healthy ewes aged 2–3 years and with 1–2 parities were selected. During the whole course of feeding (sheep house: 4.0–6.0 m^2^/sheep), each experimental sheep was fed 1.51 kg/d (dry matter intake 1.4 kg/d). During the experimental period, the feeding environment and feeding management were consistent to ensure that the sheep house was clean and that drinking water was adequate.

The embryo materials used in this experiment come from healthy Hulunbuir short-tailed sheep and Hu sheep ewes. Ewes that were artificially inseminated after natural estrus were selected, and the embryos were collected 16 and 19 days after insemination, respectively. Embryos were obtained by a surgical method, and the morphological integrity of the embryos was observed under a microscope. Samples of normally developed embryos without damage were selected. At each time point (16 days, 19 days), 3–5 Hulunbuir short-tailed sheep embryos and 3–5 Hu sheep embryos were collected from each group. This process was repeated 3 times to ensure that the sample size met the experimental requirements. All procedures were performed in accordance with animal ethical guidelines.

Primary antibody: rabbit anti-sheep monoclonal antibody (Abcam Shanghai Co., Ltd., cat. no. ab209665, 1:1000 dilution, Shanghai, China) [17]. Secondary antibody: Cy3-labeled goat anti-rabbit IgG (Wuhan Servicebio Technology Co., Ltd., cat. no. GB21303, dilution ratio 1: 300, Wuhan, China). Also used were proteinase K (Shanghai MGBio Co., Ltd., cat. no. IVD3101, Shanghai, China), eco-friendly deparaffinization solution (Wuhan Servicebio Technology Co., Ltd., cat. no. G1120), universal tissue fixative solution (Wuhan Servicebio Technology Co., Ltd., cat. no. G1101), DIG-labeled signal probe 1 (Wuhan Servicebio Technology Co., Ltd., cat. no. GDP1078), neutral gum (Sinopharm Group Co., Ltd., cat. no. PMK0429, Shanghai, China), and bovine serum albumin BSA (Wuhan Servicebio Technology Co., Ltd., cat. no. GDP1078). Other reagent consumables such as anti-fluorescence quenching sealant (Wuhan Servicebio Technology Co., Ltd., cat. no. G1401) came from Inner Mongolia Calvin Biotechnology Co., Ltd., Hohhot, China.

An optical microscope (Nikon Corporation, Shinagawa City, Japan), imaging system (Nikon Corporation, Japan), constant temperature and humidity box (Tianjin Labor Scientific Instruments Co., Ltd., Tianjin, China) dehydrating machine (DIAPATH S.p.A., Martinengo, Italy), adhesion slide (paraffin section), absolute ethanol, xylene, neutral tree glue embedding machine, freezing table, and stall machine were also used.

### 2.2. Morphological Analysis of Early Sheep Embryonic Development

#### H.E. Staining Steps

1. Paraffin sections were deparaffinized by washing with water, as follows: Sections were put into environmentally friendly deparaffinized liquid I, 20 min → environmentally friendly deparaffinized liquid II, 20 min → absolute ethanol I, 5 min → absolute ethanol II, 5 min → 75% alcohol, 5 min → water washing.

2. Pretreatment was as follows: Dyeing pretreatment solution was added for 1 min.

3. Hematoxylin staining took place as follows: Sections were stained with hematoxylin solution for 5 min → water washing → differentiation solution differentiation → water washing → return to blue solution → washing in running water.

4. Eosin staining was carried out as follows: Sections were put into 95% alcohol, 1 min → eosin staining solution, 15 s.

5. Dehydration and sealing were carried out as follows: Slicing into absolute ethanol I, 2 min → absolute ethanol II, 2 min → absolute ethanol III, 2 min → n-butyl alcohol I, 2 min → n-butyl alcohol II, 2 min → xylene I, 2 min → xylene II, 2 min → transparent → sealing.

6. Morphological observation under the microscope.

### 2.3. RNA-Level Analysis of the T Gene in Early Sheep Embryos

#### 2.3.1. Probe Methods and Repair Conditions

We used a TBXT probe set containing AACTCTCACGATGTGGATCCGAGG, TACTGGCTGTCAGCTACGTCTGTG, GGAGCATGGACAGACATGCAGATG, TCATCTCGTTGGTGAGCTCCTTGA, and TGTCTCCCGCTTCTTCCATCATCT (500 nM each).

The repair condition involved citric acid (pH 6.0) and microwaving at medium power for 7 min, and then at medium and low power for 6 min.

Digestion with proteinase K at 40 °C for 15 min.

Hybridization temperature: 40 °C.

Corresponding branch probe name: probe.

Corresponding signal probe name: DIG-labeled signal probe 1.

#### 2.3.2. SweAMI Fluorescence in Situ Hybridization on Embryonic Paraffin Sections

The SweAMI FISH technique was used, and specific oligonucleotides were tail-processed and used as probes. By using specific branching probes as intermediate hybridization media, more fluorescent signal probes can be combined to form more fluorescent labels. To achieve the localization and quantification of target gene–probe hybrids, the specific operation process includes:

1. Tissue fixation: Rinse by adding PBS → add in situ hybridization fixative → fix at 4 °C for 12 h.

2. Dehydrated embedding: Cut tissue block → low to high gradient alcohol dehydration → xylene transparent → embedded in wax.

3. Paraffin sectioning: Slicing with a slicing machine to 4 μm thick → 62 °C oven for 2 h.

4. As Step 1 of 2.2.1.

5. Repair and digestion: Histochemical circle → dropping proteinase K → digestion at 40 °C → washing with pure water → washing with PBS 3 times /5 min.

6. Prehybridization: Prehybridization solution was added and incubated at 37 °C for 1 h.

7. Hybridization: The pre-hybridization solution was discarded → add the hybridization solution containing probe → incubator hybridization overnight.

8. Wash: Discard hybridization solution → 2 × SSC, wash at 37 °C for 10 min → 1 × SSC, wash at 37 °C for 5 min → 0.5 × SSC, and wash at room temperature for 10 min.

9. Drop addition of branch probe hybridization: Dried section → drop addition of preheated branch probe, 60 μL → placed in a wet box → 40 °C, 45 min.

10. Wash: Discard hybridization solution → 2 × SSC preheated at 40 °C, 5 min → 1 × SSC, 5 min → 0.5 × SSC, 5 min → 0.1 × SSC, 5 min.

11. The corresponding signal probe was added by dropping: DIG-labeled signal probe 1 (dilution ratio 1:200) → 42 °C, 3 times, 5 min → 2 × SSC, 37 °C, 10 min → 1 × SSC, 37 °C, 2 × 5 min → 0.5 × SSC, 37 °C, 10 min.

12. DAPI counterstaining: Dropping DAPI staining solution → incubation in the dark, 8 min → washing → dropping the corresponding sealing agent.

13. Morphological observation under the microscope.

## 3. Statistics

Fluorescence intensity was quantified using ImageJ 1.8.0 (National Institutes of Health, Bethesda, MD, USA) software for relative expression. A *p* value of less than 0.05 was considered significant. Data were expressed as “mean ± standard deviation”, and a t test was used to analyze the differences between groups. *p* < 0.05 was considered statistically significant. At least three replicates were tested for each group. Prism7 software (GraphPad, Insightful Science, Boston, MA, USA) was used for statistical analysis.

## 4. Results

### 4.1. Morphological Analysis of Early Sheep Embryonic Development

**Figure 1:**
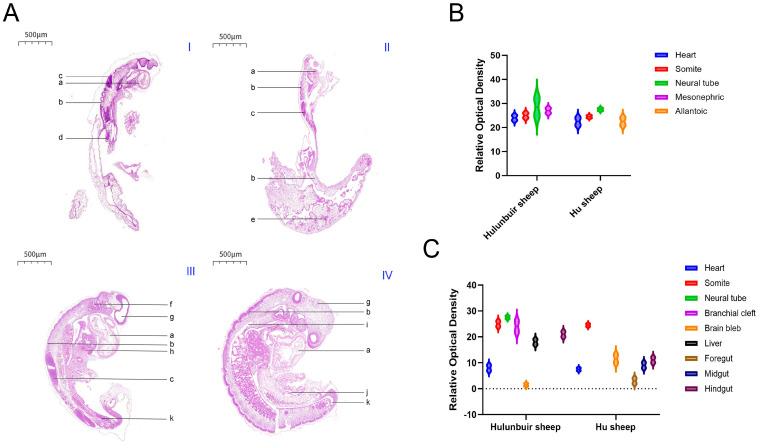
Hematoxylin–Eosin (H.E) Staining Results of Early Sheep Embryos (**AI**): H.E staining results of tissue morphology of 16-day-old Hulunbuir short-tailed sheep embryos (**AII**): H.E staining results of tissue morphology of 16-day-old Hu sheep embryos (**AIII**): H.E staining results of tissue morphology of 19-day-old Hulunbuir short-tailed sheep embryos (**AIV**): H.E staining results of tissue morphology of 19-day-old Hu sheep embryos (500 μm) (**B**): Relative optical density (ROD) of Hulunbuir sheep and Hu sheep at 16 days of embryonic age (**C**): Relative optical density (ROD) of Hulunbuir sheep and Hu sheep at 19 days of embryonic age (Key: a Heart. b Somite. c Neural tube. d Mesonephric. e Allantoic. f Branchial cleft. g Brain bleb. h Liver. i Foregut. j Midgut. k Hindgut.

At embryonic day 16, both the embryo of Hulunbuir short-tailed sheep (Figure 1(AI)) and the embryo of Hu sheep (Figure 1(AII)) showed observable morphological structures of tissues such as the heart (a), somites (b), and neural tube (c). Specifically, the embryonic structure of Hulunbuir short-tailed sheep was relatively clearer and more complete, with each labeled structure distinguishable in morphology; the Hu sheep embryo also exhibited the corresponding structures, but there were differences in overall morphological details compared with the embryo of Hulunbuir short-tailed sheep (Figure 1A).

At embryonic day 19, the embryos of Hulunbuir short-tailed sheep (Figure 1(AIII)) and Hu sheep (Figure 1(AIV)) were more well-developed, with structures including branchial clefts (f), brain vesicles (g), liver (h), foregut (i), midgut (j), and hindgut (k) appearing. The morphology and layout of various organ tissues in the embryo of Hulunbuir short-tailed sheep had their own characteristics; the Hu sheep embryo also possessed these structures, yet there were differences in morphology and tissue arrangement compared with the embryo of Hulunbuir short-tailed sheep (Figure 1A).

At embryonic day 16, there were differences of varying degrees in the relative optical density (ROD) of the heart, somites, neural tube, mesonephros, and other parts between the Hulunbuir short-tailed sheep and the Hu sheep. Among these, the ROD of the neural tube in Hulunbuir short-tailed sheep was significantly higher than that in Hu sheep, with a significant difference (*p* < 0.05), while there were also certain differences in the ROD of the heart, somites, and other parts (*p* < 0.01) (Figure 1B).

At embryonic day 19, differences in ROD were observed between Hulunbuir short-tailed sheep and Hu sheep in multiple parts including somites, branchial clefts, and brain vesicles. Specifically, the ROD of the hindgut in Hulunbuir short-tailed sheep was significantly higher than that in Hu sheep, with a significant difference (*p* < 0.05), and the ROD of the brain vesicles in Hu sheep was significantly higher than that in Hulunbuir short-tailed sheep, also with a significant difference (*p* < 0.05) (Figure 1C).

### 4.2. In Situ Hybridization Analysis of the TBXT Gene in Early Sheep Embryos

**Figure 2:**
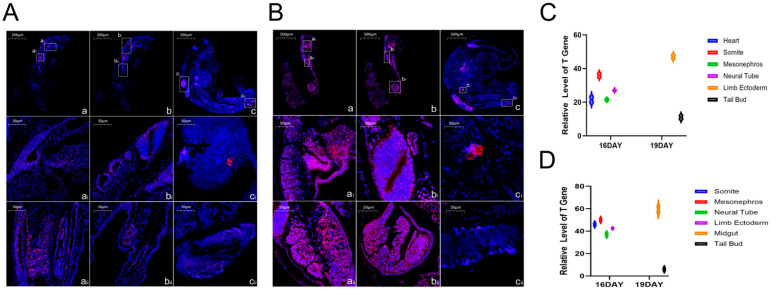
Results of In Situ Hybridization Analysis of the TBXT Gene in Early Sheep Embryos (**A**): In situ hybridization results of the TBXT gene in Hulunbuir short-tailed sheep at 16 and 19 days of embryonic age ((**A**,**B**): Panoramic diagrams of 16-day-old embryos (Scale bar: 500 μm) and 19-day-old embryos (Scale bar: 500 μm); (a(i)): Heart, (a(ii)): Somites, (b(i)): Mesonephros, (b(ii)): Neural tube, (c(i)): Limb ectoderm, (c(ii)): Tail bud; Scale bar: 50 μm); (**B**): In situ hybridization results of the TBXT gene in Hu sheep at 16 and 19 days of embryonic age ((a,b): Panoramic diagrams of 16-day-old embryos (Scale bar: 500 μm) and 19-day-old embryos (Scale bar: 500 μm); (a(i)): Heart, (a(ii)): Somites, (b(i)): Mesonephros, (b(ii)): Neural tube, (c(i)): Midgut, (c(ii)): Tail bud; Scale bar: 50 μm); (**C**): Relative RNA expression level of the TBXT gene in Hulunbuir short-tailed sheep; (**D**): Relative RNA expression level of the TBXT gene in Hu sheep.

The study presents the immunofluorescence staining results of embryonic tissues at different embryonic ages (Figure 2). The blue color represents nuclear staining (DAPI), and the red color represents the expression signal of the TBXT gene.

The results showed that: At 16 and 19 days of embryonic age, the TBXT gene was concentratedly expressed in the heart, somites, mesonephros, and neural tube of Hulunbuir short-tailed sheep and Hu sheep. Among these, the relative expression level of the TBXT gene in the heart, somites, mesonephros, and neural tube of Hulunbuir sheep was lower than that in the corresponding parts of Hu sheep, with significant differences in the relative expression level of the TBXT gene in the heart, mesonephros, and neural tube (*p* < 0.05); At 19 days of embryonic age, the TBXT gene was concentratedly expressed in the limb ectoderm and tail bud of Hulunbuir short-tailed sheep, and in the midgut and tail bud of Hu sheep. Among these, Hulunbuir sheep showed differential expression in the limb ectoderm, while Hu sheep showed differential expression in the midgut.

### 4.3. Immunofluorescence Analysis of BRACHYURY Protein in Early Sheep Embryos

**Figure 3:**
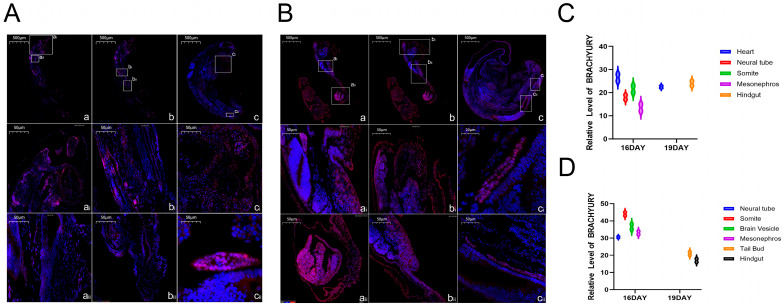
Results of Immunofluorescence Analysis of BRACHYURY Protein in Early Sheep Embryos (**A**): Immunofluorescence analysis results of BRACHYURY protein in Hulunbuir short-tailed sheep at 16 and 19 days of embryonic age ((**A**,**B**): Panoramic diagrams of 16-day-old embryos (Scale bar: 500 μm) and 19-day-old embryos (Scale bar: 500 μm); (a(i)): Heart, (a(ii)): Neural tube, (b(i)): Somites, (b(ii)): Mesonephros, (c(i)): Heart, (c(ii)): Hindgut; Scale bar: 50 μm); (**B**): Immunofluorescence analysis results of BRACHYURY protein in Hu sheep at 16 and 19 days of embryonic age ((a,b): Panoramic diagrams of 16-day-old embryos (Scale bar: 500 μm) and 19-day-old embryos (Scale bar: 500 μm); (a(i)): Neural tube, (a(ii)): Somites, (b(i)): Brain vesicles, (b(ii)): Mesonephros, (c(i)): Tail bud, (c(ii)): Hindgut; Scale bar: 50 μm); (**C**): Relative expression level of BRACHYURY protein in Hulunbuir short-tailed sheep; (**D**): Relative expression level of BRACHYURY protein in Hu sheep.

The study presents the immunofluorescence staining results of embryonic tissues at different embryonic ages (Figure 3). The blue color indicates nuclear staining (DAPI), and the red color indicates the expression signal of BRACHYURY protein.

The results showed that: At 16 days of embryonic age, BRACHYURY protein was concentratedly expressed in the neural tube, somites, brain vesicles, and mesonephros of both sheep breeds. Among these, the relative expression level of BRACHYURY protein in the neural tube, somites, and mesonephros of Hu sheep was significantly higher than that in the corresponding parts of Hulunbuir short-tailed sheep, with a significant difference (*p* < 0.05); At 19 days of embryonic age, the expression of BRACHYURY protein was concentrated in the heart and hindgut of Hulunbuir sheep, and in the tail bud and hindgut of Hu sheep. Specifically, Hulunbuir sheep showed differential expression of Brachyury protein in the heart, while Hu sheep showed differential expression in the tail bud.

## 5. Discussion

In global livestock breeding, Hulunbuir short-tailed sheep and Hu sheep are two highly recognizable, high-quality sheep breeds. With their distinct appearance and unique production advantages, they have become the core populations favored by farmers. The difference between the two sheep is particularly prominent in terms of pasture breeding. Different from the fluffy and drooping long tails of other sheep, Hulunbuir short-tailed sheep’s tails are compact and short, almost attached to the hip, which can not only reduce obstacles when moving around in grassland bushes, but also reduce the risk of frostbite on the tail in cold winters, making the sheep suitable for the complex environment of the northern grassland. Hu sheep are a “star breed” in agricultural and semi-pastoral areas around the world. As a recognized multi-parous and high-yielding sheep, they not only have a far higher reproductive efficiency than ordinary sheep, but also have a fast growth rate and strong adaptability to different climates (from warm and moist valleys to dry slopes), which makes them the best choice for farmers to achieve rapid herd expansion and efficient husbandry. The most easily distinguished feature of their appearance is the tail: it is round and oblate, and the tail tip is naturally raised, like a small pompom attached to the buttock. It not only provides the function of storing energy, but also prevents daily activities from being affected due to the excessive weight of the tail, which makes them suitable for the intensive breeding mode in farming areas.

The TBXT gene and BRACHYURYy protein play a key role in animal embryonic development. The TBXT gene is expressed in the primary streak, tail bud and other parts of the early embryo, and is involved in the regulation of mesoderm development. Its mutation or abnormal expression may lead to tail deformity. In 2018, Zhidafu found that the TBXT gene (c.G334T mutation) may be related to the abnormal tail phenotype of Hulunbuir short-tailed sheep [1]. In 2018, Day Zhipeng’s study also confirmed that the TBXT gene was highly expressed in the head and tail bud of mice, further supporting its effect on tail development [6]. As a core member of the T-box family, BRACHYURY protein is an essential component for mesoderm construction and tail formation. Lack of function of the BRACHYURY protein can lead to abnormal development of the notochord and neural tube in mouse embryos and eventually a short-tail phenotype.

At 16 and 19 days of development, the sheep embryo is at a critical stage in the formation of somites, neural tube, brain vesicles and other important structures. The gene and protein expression patterns at this stage play a decisive role in the subsequent organ differentiation and phenotype formation. However, the temporal and spatial expression differences in the TBXT gene and BRACHYURY protein between Hulunbuir and Hu sheep are not clear, and the mechanisms of their association with tail and related organ development still need to be further explored.

In this study, the embryonic development of Hulunbuir short-tailed sheep and Hu sheep at different developmental stages was explored. The results showed that the embryos of Hu sheep were slightly larger than those of Hulunbuir short-tailed sheep on the same day. According to relevant literature reports, six pairs of somites appeared in the sheep embryo on the 16th day, and the cell mass in the somites increased rapidly and showed an obvious radial arrangement. However, our H.E. staining results showed that there were seven pairs of somites in the 16-day-old Hulunbuir short-tailed sheep and Hu sheep embryos, which may be because there was a slight time difference between us and our predecessors in acquiring 16-day-old sheep embryos, or because the previous research objects were not Hulunbuir short-tailed sheep or Hu sheep. In the future, the developmental stages of embryos at different ages of the two breeds will be accurately confirmed by embryo length detection, verification of the expression levels of specific developmental marker genes, and standardized embryo staging standards, so as to eliminate the interference caused by any misjudgment of developmental stages on the statistical results of somites and further confirm the specific reasons for the differences in their number.

In terms of the development of internal organ primordia in embryos, the results of this study are in strong agreement with previous reports, which further verify the reliability of the observation. It was found that the back of the 16-day-old sheep embryos were in a zig-shaped shape, in which the foregut, midgut, hindgut and hepato-pancreatic bud could be clearly distinguished, and the ventral part of the sheep was branched from the yolk stalk to form a herringbone vitelline tube, which was completely consistent with the developmental characteristics of the digestive tract and hepato-pancreatic bud observed in the 16-day-old Hulunbuir short-tailed sheep and Hu sheep in this study. At the same time, with the development of the embryonic axis and somite, the fibrous connective tissue primordium gradually moved to the lateral side of the somite and differentiated into mesenchymal cells of the dermoderm. These cells were syncytial-like and extended ventrally to the ectoderm, and the cell processes were connected to each other. The neural tube appeared as a long oblate cone, which was thick in front and thin in the back, and was curved to form an arcuate spinal primordium. The branchial cleft gradually degenerated, and the primary opening communicated with the foregut. In the embryo, the foregut, midgut and hindgut still maintain a straight tubular shape, and the ventral part of the midgut leads directly to the yolk sac. The development rate of the intestine is accelerated, which leads to the differentiation of the foregut, midgut and hindgut at the posterior end of the gastric bud to form a V-shaped long tube. The developmental characteristics of the internal structure of the above embryos are highly consistent with the results reported in the literature, providing reliable basic data support for the subsequent in-depth study of the differences in embryo development between the two breeds [18].

SweAMI FISH fluorescence in situ hybridization analysis of the 16- and 19-day-old Hulunbuir short-tailed sheep and Hu sheep embryos, combined with the conserved functions of the TBXT gene in regulating mesoderm differentiation, body axis formation, and organ rudiment development, identified its key regulatory role in embryonic development in the two varieties: the TBXT gene was mainly expressed in the heart, somite, neural tube and mesonephros in the 16-day-old embryos of both varieties, and played a multidimensional regulatory function. We inferred that the TBXT gene could promote the differentiation of mesodermal cells into somites and ensure the stable formation and orderly arrangement of the seven pairs of somites in the 16-day-old embryos. This lays the foundation of the adult spine structure and trunk length (such as the short tail characteristic of Hulunbuir short-tailed sheep or the regulation of the tail somite by this gene) and provides a framework for the positioning and development of limbs and internal organs. In the heart and circulatory system, TBXT regulates the proliferation and differentiation of cardiac mesoderm cells to promote the formation of the heart primordium and the establishment of the early circulatory system, and ensures the growth of the 16- and 19-day-old embryos (the Hu sheep embryo is slightly larger, which could be related to the effect of the expression intensity in the heart on nutrient supply). In the neural tube and nervous system, TBXT regulates the proliferation and migration of neural tube cells and the establishment of the dorsoventral axis, assists in the differentiation of brain blebs and spinal cord primordium and the formation of early neural circuits, and affects subsequent neurological function. In terms of the mesonephros, excretory and reproductive systems, TBXT is involved in the differentiation of mesonephros tubules to establish excretory functions and may also indirectly affect the development of the reproductive system, laying the foundation for the multiple pregnancies characteristic of Hu sheep. The distribution of the gene in Hulunbuir sheep embryos was different from that in the control group. In Hu sheep embryos, TBXT is highly expressed in the midgut and tail bud. The former may promote the development of the gut to adapt to efficient digestion (fitting the characteristics of rapid growth), and the latter may maintain cell proliferation to form a flat upsloping tail, and the development of the midgut may help in subsequent nutrient reserves. In zebrafish (*Danio rerio*), recent studies continue to focus on the TBXT homolog ntl (no tail). ntl genes are not only essential for notochord development and body axis elongation, but also involved in cell fate determination and tissue morphogenesis. Using CRISPR/Cas9-mediated gene editing technology to construct ntl mutants, it was found that the cell migration and differentiation were abnormal during gastrula formation, resulting in severe retardation of mesoderm and notochord development, which affected the normal body axis construction [19]. The key role of the TBXT homolog Xbra in mesoderm induction and pattern formation has also been identified in Xenopus laevis. Using RNA sequencing and ChIP-seq technologies, we found that Xbra activates the expression of a series of downstream genes related to mesoderm differentiation and body axis development by binding to specific cis-regulatory elements, forming a complex transcriptional regulatory network. During gastrulation, Xbra dynamically regulates cell motion-related genes to ensure the correct migration and differentiation of mesodermal cells and maintain the normal development of embryos [20]. In Homo sapiens, the TBXT gene is closely related to embryonic development and disease occurrence. A study published in Nature in 2024 found that ape-like and human-specific Alu elements were inserted into the intron of the TBXT gene, resulting in alternative splicing of the gene and producing shorter transcripts, which in turn affected tail development, providing a genetic explanation for the disappearance of the tail in humans and apes [21].

The TBXT gene not only ensures the basic embryonic development of the two varieties in the co-expression region, but also realizes differential regulation through specific expression at 19 days old, which becomes an important molecular link for the differences in adult body size and performance between the two varieties. The mechanism can be further analyzed by combining the expression intensity and downstream target genes. In addition, by observing the details of the tail of 19-day-old Hulunbuir short-tailed sheep embryos, we found that the positive position of the tail staining was deeper and more specific, which was consistent with the results of in situ hybridization of early mouse embryos. TBXT was first found to be highly expressed in the midgut of the 19-day-old Hu sheep embryo. Combined with the conserved functions of this gene in regulating mesoderm differentiation, organ primordium development and body axonalization, we speculated that the TBXT gene could play a multifunctional role in regulating the development of the Hu sheep gut; by regulating the proliferation rate of mesodermal cells in the midgut wall, it provides a cell basis for the thickening of the midgut wall and the elongation of the intestine, which is suitable for the rapid growth demands of Hu sheep embryos in terms of intestinal expansion [22]. It participates in the regulation of midgut mesoderm differentiation into smooth muscle cells, promotes the formation of the intestinal smooth muscle primordium, and lays the structural foundation for the subsequent establishment of intestinal peristalsis function. It mediates the interaction between the midgut and adjacent tissues (such as the yolk sac and neural tube) to clarify the spatial location of the midgut in the embryo and ensure the coordinated development of the gut and other organs (such as hepatopangeal buds). It also regulates the junction and differentiation of midgut epithelial cells and mesodermal cells, promotes the early formation of intestinal villus primordium, and lays the groundwork for the efficient nutrient absorption capacity of adult Hu sheep. It is involved in the regulation of midgut bending and rotation and promotes the transformation of the midgut from straight and tubular to a complex intestinal structure (with features such as intestinal loops) by affecting the direction of cell migration of the midgut tissue. It regulates the activity of mesoderm-derived mesenchymal cells, affects the early development of the intestinal lamina propria, and provides a microenvironment for the subsequent colonization of intestinal immune-related cells. By regulating the mesodermal signaling pathway, it induces the differentiation of epithelial cells into cell types with digestive function, which provides support for the formation of autonomous digestion ability in the later embryonic stages of Hu sheep. The above predictions are highly consistent with the characteristics of Hu sheep with fast growth and high digestion efficiency. In the future, we will conduct further research in this direction. At present, our study visually demonstrates the expression location and quantity of TBXT genes in Hulunbuir and Hu sheep embryos on Day 16 and Day 19 at the RNA level, complementing the data gaps at the transcriptome level and regarding the spatial location of the TBXT gene in early sheep embryos.

The key role of Brachyury protein (encoded by the TBXT gene) in the embryonic development of Hulunbuir and Hu sheep can be clarified by combining related research reports and the expression characteristics of Brachyury protein. Previous studies have shown that the TBXT gene can cooperate with other proteins to activate specific signaling pathways to guide embryonic development [6], and its function is closely related to the Cdx gene. Amin et al. found that the Cdx gene is essential for the development of the anterior and posterior axes of mouse embryos, and Cdx mutant embryos cannot elongate the posterior axis normally. This is consistent with the TBXT gene defect phenotype [23]. At the same time, Su et al. obtained short-tailed mice by editing the TBXT gene through CRISPR/Cas9 [24], and Xia B et al. also proposed that the mutation of this gene was the main reason for the difference in tail phenotype between humans and apes, which confirmed that BRACHYURY protein plays a central role in the regulation of anterior and posterior axes of embryos and the formation of the short-tailed phenotype [21]. According to the expression of this protein, it was concentrated in the ganglia, somites and brain vesicles of the two varieties of embryos at the embryonic age of 16 days, suggesting that it regulates nerve cell differentiation and ganglion formation through signaling pathways in the ganglia to lay the foundation of early neural function. In the somites, it cooperates with Cdx genes to regulate the arrangement and differentiation of somites along the anteroposterior axis to construct the body’s structural framework and lay a species-specific basis for tail development. In the brain vesicles, it participates in the differentiation of the brain primordium to help in the development of the central nervous system. On the 19th day, its expression was breed-specific; it was expressed in the heart and hindgut of Hulunbuir sheep embryos, which was speculated to regulate the maturation of cardiomyocytes and the improvement of cardiac structure to ensure circulation function in the heart, and it was involved in the differentiation of the intestinal terminal and indirectly enhanced the short-tail phenotype through related signals combined with the short-tail characteristics in the hindgut. It was expressed in the tail bud and hindgut of the Hu sheep embryo. The expression in the tail bud can maintain cell proliferation activity to form a flat tail, and the expression in the hindgut is involved in the differentiation of digestive cells at the end of the intestine to adapt to its characteristics of fast growth and high digestion efficiency. Therefore, we concluded that BRACHYURY protein ensured the core development process of the two breeds’ embryos through its general expression at Day 16, while its specific expression at Day 19, combined with its core function, contributed to the tail and gut development of Hulunbuir sheep and Hu sheep, respectively. It therefore became an important regulatory factor for the formation of differences in adult characteristics and performance between the two varieties.

By detecting the expression differences in the TBXT gene and BRACHYURY protein in 16-day-old and 19-day-old sheep embryos, the spatiotemporal distribution characteristics of the TBXT gene and Brachyury protein in the early sheep embryo were visualized at the RNA and protein levels for the first time, which complemented the data gaps in the transcriptome and spatial localization of the TBXT gene in sheep embryos. This study provides a new experimental basis for understanding the molecular mechanisms of sheep embryonic development. Understanding the differences in the expression of the TBXT gene and BRACHYURY protein between the two kinds of sheep will help to reveal the differences in molecular regulation pathways of tail formation between Hulunbuir and Hu sheep and provide key clues for elucidating the genetic mechanisms of sheep tail development. These results provide a theoretical basis for the genetic analysis of sheep-breed-specific traits (such as tail morphology and multi-fetal characteristics) and an experimental basis for screening molecular markers related to sheep tail development and breed improvement.

## 6. Conclusions

There are differences in the temporal and spatial distribution of the TBXT gene and BRACHYURY protein expression between Hulunbuir and Hu sheep, suggesting that the molecular regulation pathways of the tail and related organ development may be different between the two breeds. This study provides an important theoretical basis for the mechanisms of genetic differences in sheep tail development.

## Data Availability

Data are contained within the article.
